# Impact of resistance exercise on ribosome biogenesis is acutely regulated by post‐exercise recovery strategies

**DOI:** 10.14814/phy2.12670

**Published:** 2016-01-27

**Authors:** Vandré C. Figueiredo, Llion A. Roberts, James F. Markworth, Matthew P. G. Barnett, Jeff S. Coombes, Truls Raastad, Jonathan M. Peake, David Cameron‐Smith

**Affiliations:** ^1^ The Liggins InstituteThe University of AucklandAucklandNew Zealand; ^2^ School of Human Movement and Nutrition SciencesThe University of QueenslandBrisbaneAustralia; ^3^ Centre of Excellence for Applied Sport Science ResearchQueensland Academy of SportBrisbaneAustralia; ^4^ AgResearch LimitedGrasslands Research CentrePalmerston NorthNew Zealand; ^5^ Norwegian School of Sport SciencesOsloNorway; ^6^ School of Biomedical Sciences and Institute of Health and Biomedical InnovationQueensland University of TechnologyBrisbaneAustralia

**Keywords:** Cyclin D1, hypertrophy, pre‐rRNA, ribosomal RNA, skeletal muscle, upstream binding factor

## Abstract

Muscle hypertrophy occurs following increased protein synthesis, which requires activation of the ribosomal complex. Additionally, increased translational capacity via elevated ribosomal RNA (rRNA) synthesis has also been implicated in resistance training‐induced skeletal muscle hypertrophy. The time course of ribosome biogenesis following resistance exercise (RE) and the impact exerted by differing recovery strategies remains unknown. In the present study, the activation of transcriptional regulators, the expression levels of pre‐rRNA, and mature rRNA components were measured through 48 h after a single‐bout RE. In addition, the effects of either low‐intensity cycling (active recovery, ACT) or a cold‐water immersion (CWI) recovery strategy were compared. Nine male subjects performed two bouts of high‐load RE randomized to be followed by 10 min of either ACT or CWI. Muscle biopsies were collected before RE and at 2, 24, and 48 h after RE. RE increased the phosphorylation of the p38‐MNK1‐eIF4E axis, an effect only evident with ACT recovery. Downstream, cyclin D1 protein, total eIF4E, upstream binding factor 1 (UBF1), and c‐Myc proteins were all increased only after RE with ACT. This corresponded with elevated abundance of the pre‐rRNAs (45S, ITS‐28S, ITS‐5.8S, and ETS‐18S) from 24 h after RE with ACT. In conclusion, coordinated upstream signaling and activation of transcriptional factors stimulated pre‐rRNA expression after RE. CWI, as a recovery strategy, markedly blunted these events, suggesting that suppressed ribosome biogenesis may be one factor contributing to the impaired hypertrophic response observed when CWI is used regularly after exercise.

## Introduction

Resistance exercise (RE) training results in marked structural and contractile adaptations within skeletal muscle. This complex tissue remodeling is dependent upon increased protein synthesis to enable muscle fiber hypertrophy. The ribosome is the cellular machinery that translates the transcriptional gene information of the messenger RNA (mRNA) into new proteins. In addition to ribosomal activation (translation initiation), recent evidence has implicated ribosome biogenesis in the dynamic regulation of skeletal muscle mass (von Walden et al. 2012; Figueiredo et al. 2015; Kirby et al. 2015), with increased transcription of the ribosomal DNA (rDNA) into ribosomal RNA (rRNA). However, little is known of the activation of mechanisms governing ribosome biogenesis and the time course of rDNA transcription following a bout of RE.

The first and rate‐limiting step of ribosome biogenesis is transcription of the rDNA into the precursor 45S rRNA (Panov et al. 2001). This requires various transcriptional factors such as upstream binding factor (UBF) and transcription initiation factor IA (TIF‐IA) to bind to the rDNA promoter and induce the synthesis of the pre‐rRNA by the dedicated RNA polymerase I (Pol I) (Moss et al. 2007). After 45S pre‐rRNA is transcribed, it is rapidly processed by cleavage into multiple transcripts which form the mature rRNAs, 28S, 18S, and 5.8S. Together with 80+ ribosomal proteins by RNA polymerase II and 5S rRNA transcribed by rRNA polymerase III, these mature rRNAs eventually form the large and small subunits of the ribosome (Henras et al. 2015).

The regulation of UBF protein activity is central for rRNA synthesis and cell growth (Voit et al. 1995, 1999; Drakas et al. 2004). Its phosphorylation status is a key determinant for rDNA transcription (Voit et al. 1995; Voit and Grummt 2001). UBF is phosphorylated by cyclin‐dependent kinase 4 (CDK4) when a complex is formed between CDK4 and the cell cycle regulator, cyclin D1. We have recently proposed that high‐load‐resistance exercise increases cyclin D1 protein expression in skeletal muscle by specifically targeting the translation of cyclin D1 mRNA (Figueiredo et al. 2015) through activation of the MAP kinase interacting serine/threonine kinase 1 (MNK1) and eukaryotic translation initiation factor 4E (eIF4E) (Topisirovic et al. 2004).

In the current study, ribosome biogenesis was examined from 2 h post exercise and extending to 24 h and 48 h post‐RE in healthy trained subjects. Differing recovery strategies following intense RE are widely used by athletes, including cold‐water immersion. While acute cold‐water immersion (CWI) has been shown to provide analgesia and reduce delayed onset muscle soreness (Bleakley et al. 2012) and muscle edema (Yanagisawa et al. 2003), it may also negatively affect long‐term muscle adaptation, as determined by training‐induced muscle hypertrophy and strength gains (Roberts et al. 2015; Yamane et al. 2015). Thus, it was hypothesized that CWI would suppress the transcriptional signaling and activation of rRNA synthesis during exercise recovery.

## Methods

### Subjects and study design

The participant characteristics and study design are described in detail elsewhere (Roberts et al. 2015). All participants were informed of the requirements and risks of the study in addition to be provided with a written informed consent. The experimental procedures were approved by the Human Research Ethics Committee of The University of Queensland. Nine recreationally active young males (22.1 ± 2.2 years old), with at least 1‐year experience in resistance training, completed two single‐leg resistance training sessions on separate days. These sessions comprised 45° leg press, single‐leg squats, knee extensions, and walking lunges. These exercises involved performing 3–6 sets until failure, with loads of 8, 10, and 12 repetitions maximum (RM). In a randomized, cross‐over design study, the participants completed one of two recovery treatments, beginning 5 min after exercise. These treatments involved cold‐water immersion (CWI) or active recovery (ACT). For the CWI trial, the participants sat up to their waist in cold water (10.0 ± 0.3°C) contained in an inflatable bath (iBody; iCool, Miami, QLD, Australia) for 10 min. In the ACT trial, the participants cycled on a stationary bicycle (Wattbike, Nottingham, U.K.) at a low, self‐selected intensity (36.6 ± 13.8) for 10 min. Muscles biopsies from the *Vastus lateralis* were collected before and 2, 24, and 48 h after each session. Muscle samples were snap frozen in liquid nitrogen and stored at −80°C until further analysis.

The phosphorylation status and abundance of proteins involved in ribosome biogenesis were assessed by Western blot. A total of 25 mg of tissue was homogenized in RIPA lysis buffer (Millipore, Temecua, CA) supplemented with Halt^TM^ protease and phosphatase inhibitor cocktail (Thermo Scientific, Waltham, MA). Samples were centrifuged, and the supernatant was used to measure total protein concentration using a Pierce^™^ BCA Protein Assay Kit (Thermo Scientific). Equal amounts of protein were boiled in 4× Laemmli buffer; 20 *μ*g of protein was separated by SDS‐PAGE and then transferred to PVDF membranes (Bio‐Rad Laboratories, Inc., Hercules, CA) using a semidry Trans‐Blot Turbo^™^ device (Bio‐Rad). Following 1 h blocking with 5% bovine serum albumin solution in Tris‐buffered saline with 0.1% Tween 20 (TBST), the membranes were incubated with primary antibodies (1:1000 dilution, with exception of GAPDH which was 1:10,000) overnight at 4°C. The following antibodies were used in this study: total UBF (#13125), p‐UBF Ser484 (#21638) and Ser388 (#21637), cyclin D1 (#450), total eIF4E (#9976) (Santa Cruz Biotechnology, Santa Cruz, CA), total TIF‐IA (#42539), p‐TIF Ser649 (#138651), GAPDH (#36840)(Abcam, Cambridge, U.K.), total Akt (#2920), p‐Akt Thr308 (#4056), total PRAS40 (#2691), p‐PRAS40 Thr246 (#2691), p‐p38 MAPK Thr180/Tyr182 (#4511), p‐eIF4E Ser209 (#9741), p‐MNK1 Thr197/202 (#2111)(Cell Signaling Technology, Inc., Danvers, MA), and total c‐Myc – 9E10 clone (Developmental Studies Hybridoma Bank, Iowa City, IA). The next day, the membranes were washed in TBST before they were incubated with the appropriate anti‐rabbit or anti‐mouse secondary antibodies (Jackson ImmunoResearch Laboratories, West Grove, PA) linked to horseradish peroxidase (1:5000) for 1 h at room temperature. The membranes were once again washed in TBST and exposed on a ChemiDoc image device (Bio‐Rad) using enhanced chemiluminescence reagent (ECL Select kit; GE Healthcare Ltd., Little Chalfont, U.K.). Bands were quantified using ImageJ software (NIH, Bethesda, MD).

Total RNA was extracted from ~20 mg of muscle tissue using the AllPrep^®^ DNA/RNA/miRNA Universal Kit (QIAGEN GmbH, Hilden, Germany) following the manufacturer's instructions. Following cDNA synthesis using High‐Capacity RNA‐to‐cDNA^™^ kit (Life Technologies, Carlsbad, CA), messenger and ribosomal RNA (mRNA and rRNA) were measured by RT‐PCR on a LightCycler 480 II (Roche Applied Science, Penzberg, Germany) using SYBR Green I Master Mix (Roche Applied Science). Target mRNAs were UBF (*UBFT*), polymerase RNA I polypeptide B (*POLR1B*), TIF‐IA (*RRN3*), and cyclin D1 (*CCND1*). Pre‐rRNAs were measured using primers designed specifically for pre‐rRNA sequences spanning the 5′‐end internal/external transcribed spacer (ITS/ETS) region, and a specific primer for the initial external transcribed spacer (ETS) region of 5′‐end of 45S rRNA, namely ITS‐5.8S, ITS‐28S, ETS‐18S, and 45S. These primers detect only the pre‐rRNAs, which after processing and maturation will form mature ribosomes (Henras et al. 2015). Primers for the internal region of the mature rRNAs (28S, 18S, and 5.8S) were also used. Primers for rRNAs were designed by QIAGEN, using RT^2^ Profiler PCR Arrays (QIAGEN). We have previously described these primers and some target mRNAs applied in this study (Figueiredo et al. 2015). The sequences for the other targets not described before are shown in Table 1. The geometric mean of three reference genes (Vandesompele et al. 2002) was used for normalization. The recently proposed human reference genes (Eisenberg and Levanon 2013), chromosome 1 open reading frame 43 (*C1orf43*), charged multivesicular body protein 2A (*CHMP2A*), and ER membrane protein complex subunit 7 (*EMC7*) were identified as the least variable and were, therefore, used as reference genes. Standard and melting curves were performed for every target to confirm primer efficiency and single‐product amplification.

**Table 1:** RT‐PCR primer sequences

Target	Primer sequence
*EMC7*, forward	GGGCTGGACAGACTTTCTAATG
*EMC7*, reverse	CTCCATTTCCCGTCTCATGTCAG
*CHMP2A*, forward	CGCTATGTGCGCAAGTTTGT
*CHMP2A*, reverse	GGGGCAACTTCAGCTGTCTG
*C1orf43*, forward	CTATGGGACAGGGGTCTTTGG
*C1orf43*, reverse	TTTGGCTGCTGACTGGTGAT
*NIP7*, forward	CCGGGTGTACTATGTGAGTGAGAA
*NIP7*, reverse	TTGTGGGTTTTAGTGAATTTTCCA

### Statistical analysis

RT‐PCR data were analyzed using the 2^−∆∆CT^ method. Western blot data are presented as fold change against the protein expression for the pre‐exercise sample for each trial. Data are expressed as mean ± standard error of the mean (SEM). Main effects of time and trial and time × trial interactions were assessed using a two‐way ANOVA (time post exercise × recovery strategy) with repeated measures (SigmaPlot v12.0, Systat Software, Inc., San Jose, CA). Student–Newman–Keuls post hoc tests were used to determine the significance of pair‐wise comparisons of changes with time and differences between the trials. Statistical significance was accepted at *P *≤* *0.05.

## Results

### Effect of resistance exercise and recovery strategies on protein response

#### p38 MAPK

**Figure 1:**
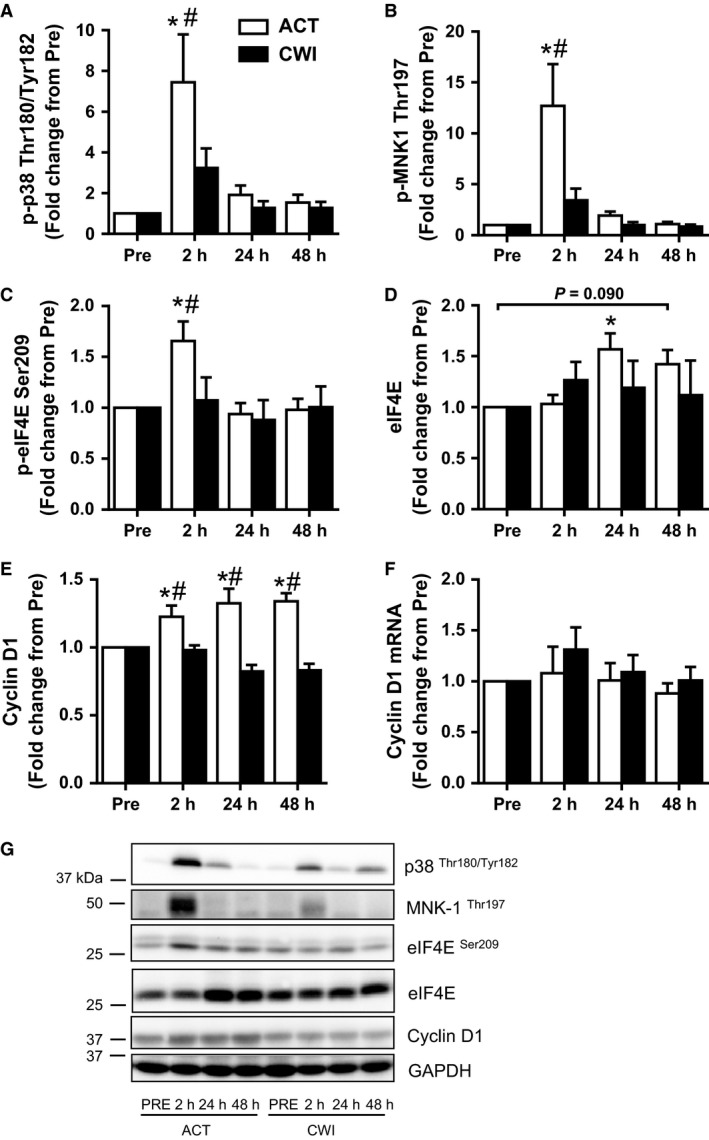
Effect of resistance exercise on the p38‐MNK1‐eIF4E axis. Phosphorylation levels of p38 (A), MNK1 (B), and eIF4E (C), and total levels of eIF4E (D), total cyclin D1 protein (E), and mRNA levels (F). Representative Western blot figures (closest molecular weight marker is shown in the left‐hand side) (G). Western blot data were normalized to GAPDH, with the exception of eIF4E Ser209, which was normalized to its respective total protein. Cyclin D1 mRNA expression was normalized to the geometric mean of three reference genes. Values are mean ± SEM. *different from PRE within the same trial (*P* < 0.05), ^#^different between trials within the same time point (*P* < 0.05). Main effects and interactions are presented in the text.

Phosphorylation of p38 at the dual site Thr180/Tyr182 (p‐p38 MAPK Thr180/Tyr182) increased after exercise (time effect *P* < 0.001), and this response tended to differ between the trials (interaction *P* = 0.068) (Fig. 1A). In the ACT trial, p‐p38 MAPK Thr180/Tyr182 was increased 2 h post exercise (*P* < 0.001), while it was not statistically different at the same time point (*P* = 0.25) in the CWI trial. Compared with the CWI trial, p‐p38 MAPK Thr180/Tyr182 was higher 2 h post exercise in the ACT trial (*P* = 0.003).

#### MNK1

Phosphorylation of MNK1 at Thr197 (p‐MNK1 Thr197) increased after exercise (time effect *P* < 0.001) and was different between the trials (interaction *P* = 0.01) (Fig. 1B). p‐MNK1 Thr197 was only higher than pre‐exercise 2 h after exercise (*P* < 0.001) in the ACT trial. Compared with the CWI trial, it was higher 2 h after exercise in the ACT trial (*P* < 0.001).

#### eIF4E

Phosphorylation of eIF4E at Ser209 (p‐eIF4E Ser209) followed a similar pattern to its upstream kinase, MNK1. p‐eIF4E Ser209 increased after exercise (time effect *P* = 0.021) and this response tended to differ between the trials (interaction *P* = 0.074) (Fig. 1C). p‐eIF4E Ser209 was higher than pre‐exercise 2 h after exercise (*P* = 0.001) in the ACT trial. It was also higher at this time after the ACT trial compared with the CWI trial (*P* = 0.009). Total eIF4E protein expression also increased after exercise (time effect *P* = 0.043) (Fig. 1D). It was higher than pre‐exercise 24 h post‐exercise (*P* = 0.028) and tended to remain higher 48 h post exercise (*P* = 0.090) only in the ACT trial (Fig. 1D).

#### Cyclin D1

The change in cyclin D1 total protein expression was different between the trials (interaction *P* < 0.001) (Fig. 1E). It was higher than pre‐exercise 2 h (*P* = 0.002), 24 h (*P* < 0.001), and 48 h (*P* < 0.001) after exercise in the ACT trial. It was also higher at all three time points after the ACT trial compared with the CWI trial: 2 h (*P* = 0.012), 24 h (*P* < 0.001), and 48 h (*P* < 0.001).

#### Akt

**Figure 2:**
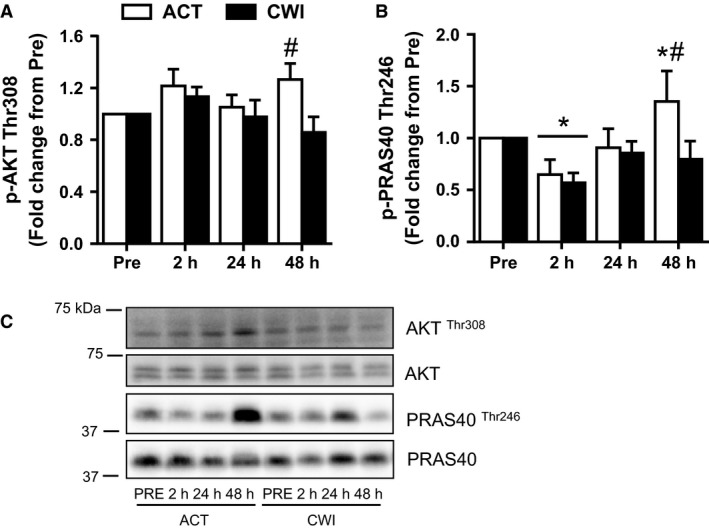
Effect of resistance exercise on Akt signaling. Phosphorylation levels of AKT (A) and PRAS40 (B). Representative Western blot figures (closest molecular weight marker is shown in the left‐hand side) (C). Western blot data were normalized to their respective total proteins. Values are mean ± SEM. *different from PRE within the same trial (*P* < 0.05); ^#^different between trials within the same time point (*P* < 0.05). Main effects and interactions are presented in the text.

The change in phosphorylation of Thr308 on Akt was different between the trials (interaction *P* = 0.047) (Fig. 2A). Compared with the CWI trial, it was higher 48 h (*P* = 0.005) after exercise in the ACT trial.

#### PRAS40

Phosphorylation of Thr246 on PRAS40 (p‐PRAS40 Thr246) changed after exercise (time effect *P* = 0.002) (Fig. 2B). It was lower than pre‐exercise 2 h after exercise in the ACT trial (*P* = 0.005) and the CWI trial (*P* = 0.005). It was higher than pre‐exercise 48 h after exercise in the ACT trial (*P* = 0.043). Compared with the CWI trial, p‐PRAS40 Thr246 was higher 48 h (*P* = 0.011) after exercise in the ACT trial.

#### UBF

**Figure 3:**
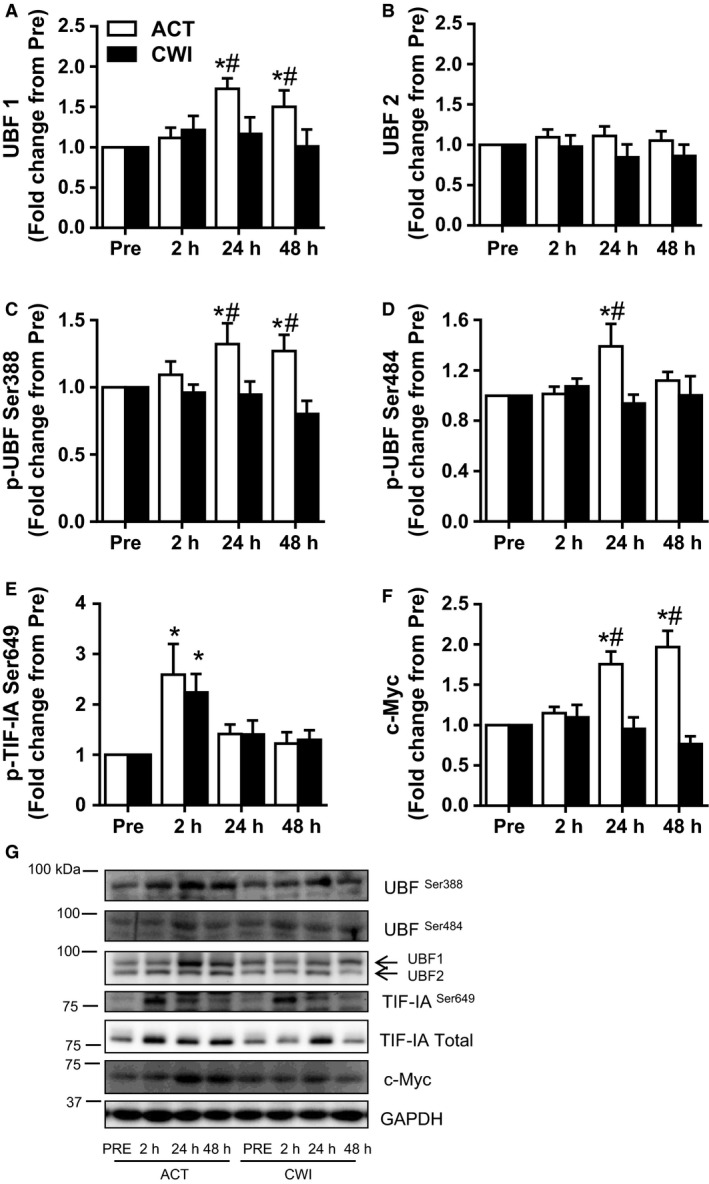
Effect of resistance exercise on rDNA transcription factors. Protein levels of UBF1 (A), UBF2 (B), phosphorylation of UBF at Ser388 (C) and Ser484 (D), TIF‐IA at Ser649 (E), and protein levels of c‐Myc (F). Representative Western blots figures (closest molecular weight marker is shown in the left‐hand side) (G). Western blot data were normalized to GAPDH. Values are mean ± SEM. *different from PRE within the same trial (*P* < 0.05); ^#^different between trials within the same time point (*P* < 0.05). Main effects and interaction are presented in the text.

UBF1 total protein expression increased after exercise (time effect *P* = 0.028), and this response was different between the trials (interaction *P* = 0.041) (Fig. 3A). It was higher than pre‐exercise 24 h (*P* = 0.004) and 48 h (*P* = 0.040) after exercise in the ACT trial. Compared with the CWI trial, UBF1 total protein was higher 24 h (*P* = 0.022) and 48 h (*P* = 0.015) after exercise in the ACT trial. UBF2 total protein expression did not change significantly after exercise (time effect *P* = 0.63) in either trial (Fig. 3B).

UBF phosphorylation at Ser388 (p‐UBF Ser388) followed the same pattern as UBF1 total protein (Fig. 3C). The change in p‐UBF Ser388 was different between the trials (interaction *P* = 0.030). It was higher than pre‐exercise 24 h (*P* = 0.018) and 48 h (*P* = 0.034) after exercise in the ACT trial. Compared with the CWI trial, p‐UBF Ser388 was higher 24 h (*P* = 0.015) and 48 h (*P* = 0.007) after exercise in the ACT trial. The change in p‐UBF Ser484 was also different between the trials (interaction *P* = 0.032) (Fig. 3D). It was higher than pre‐exercise 24 h (*P* = 0.006) after exercise in the ACT trial. Compared with the CWI trial, p‐UBF Ser484 was also higher 24 h (*P* = 0.006) after exercise in the ACT trial.

#### TIF‐IA

Phosphorylation of Ser649 on TIF‐IA increased after exercise (time effect *P* = 0.006) (Fig. 3E). It was higher than pre‐exercise 2 h after exercise in both the ACT (*P* = 0.046) and CWI (*P* = 0.002) trials.

#### c‐Myc

The change in c‐Myc total protein expression was different between the trials (interaction *P* < 0.001) (Fig. 3F). It was higher than pre‐exercise 24 h (*P* < 0.001) and 48 h (*P* < 0.001) after exercise in the ACT trial. Compared with the CWI trial, c‐Myc total protein was higher 24 h (*P* < 0.001) and 48 h (*P* < 0.001) after exercise in the ACT trial.

### Effect of resistance exercise and recovery strategies on rRNA and mRNA expression

#### rRNA

**Figure 4:**
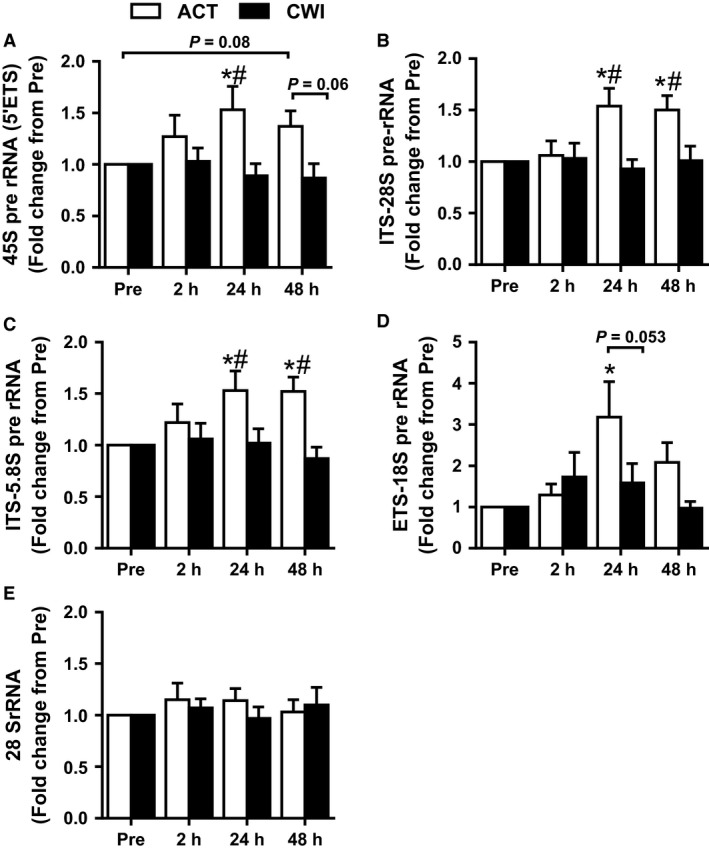
Effect of resistance exercise on rDNA transcription. Expression of pre‐rRNA as detected by primers against: 5′ETS (A), ITS‐5.8S (B), ITS‐28S (C), ETS‐18S (D), and mature 28S rRNA (E). rRNA expression was normalized by geometric mean of three reference genes. Values are mean ± SEM. *different from PRE within the same trial (*P* < 0.05); ^#^different between trials within the same time point (*P* < 0.05). Main effects and interaction are presented in the text.

45S pre‐rRNA (5′ETS) was increased after 24 h in the ACT trial (*P* = 0.018) and tended to remain elevated after 48 h (*P* = 0.089) (Fig. 4A). 45S pre‐rRNA did not change significantly in the CWI trial. 45S pre‐rRNA was higher 24 h (*P* = 0.035) after exercise and tended to remain higher 48 h (*P* = 0.065) after exercise in the ACT trial compared with the CWI trial. ITS‐28S rRNA was different between the trials (interaction *P* = 0.010) (Fig. 4B). It was higher than pre‐exercise 24 h (*P* = 0.003) and 48 h (*P* = 0.003) after exercise in the ACT trial. Compared with the CWI trial, it was higher 24 h (*P* = 0.007) and 48 h (*P* = 0.016) after exercise in the ACT trial. The change in ITS‐5.8S rRNA was also different between the trials (interaction *P* = 0.032) (Fig. 4C). It was higher than pre‐exercise 24 h (*P* = 0.007) and 48 h (*P* = 0.004) after exercise in the ACT trial. Compared with the CWI trial, it was higher 24 h (*P* = 0.050) and 48 h (*P* = 0.005) after exercise in the ACT trial. ETS‐18S pre‐rRNA was only increased in the ACT trial at 24 h (*P* = 0.004), and it showed a trend to be higher compared to CWI trial at the same time point (*P* = 0.053) (Fig. 4D). The mature 28S rRNA did not change significantly after either trial (time effect *P* = 0.73) (Fig. 4E). Further, mature 18S and 5.8S followed the same response and were unchanged at all time points (data not shown).

#### Cyclin D1 mRNA

There were no significance changes over time or differences between trials (time effect *P* = 0.41, interaction *P* = 0.80) for cyclin D1 mRNA (Fig. 1F).

#### UBF mRNA

**Figure 5:**
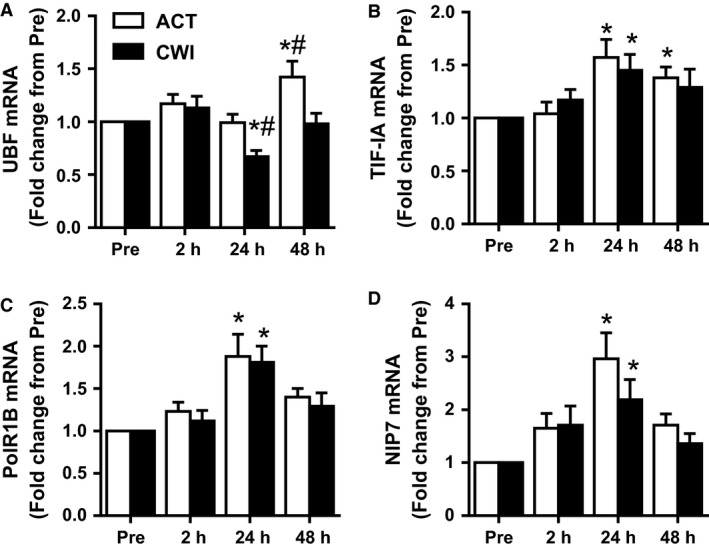
Effect of resistance exercise on mRNA levels of genes involved in rDNA transcription. Expression of UBF (A), TIF‐IA (B), PolR1B (C), NIP7 (D) mRNAs. mRNA expression was normalized by geometric mean of three reference genes. Values are mean ± SEM. *different from PRE within the same trial (*P* < 0.05); ^#^different between trials within the same time point (*P* < 0.05). Main effects and interaction are presented in the text.

UBF mRNA increased after exercise (time effect *P* < 0.001), and this response tended to differ between the trials (interaction *P* = 0.080) (Fig. 5A). It was higher than pre‐exercise 48 h after exercise (*P* = 0.006) in the ACT trial, whereas it was lower than pre‐exercise 24 h after exercise (*P* = 0.037) in the CWI trial. Compared with the CWI trial, UBF mRNA was higher 24 h (*P* = 0.040) and 48 h (*P* = 0.007) after exercise in the ACT trial.

#### TIF‐IA mRNA

TIF‐1A mRNA increased after exercise (time effect *P* < 0.001) (Fig. 5B). It was higher than pre‐exercise 24 h after exercise in the ACT trial (*P* = 0.004) and CWI trial (*P* = 0.026) and remained above pre‐exercise 48 h after exercise in the ACT trial (*P* = 0.048).

#### PolR1B mRNA

PolR1B mRNA also increased after exercise (time effect *P* < 0.001) (Fig. 5C). It was higher than pre‐exercise 24 h after exercise in both the ACT (*P* = 0.001) and CWI (*P* = 0.033) trials.

#### NIP7 mRNA

NIP7 mRNA increased after exercise (time effect *P* < 0.001) (Fig. 5D). It was also higher than pre‐exercise 24 h after exercise in both the ACT trial (*P* < 0.001) and CWI trial (*P* = 0.015), with no significant differences between trials.

## Discussion

Ribosome biogenesis has recently been implicated in muscle hypertrophy induced by RE (Figueiredo et al. 2015). In the current study, a single bout of high‐load RE activated multiple cellular signaling events that enable the activation of transcriptional factors involved in the recruitment of Pol I, the initiation of ribosome biogenesis and increased pre‐rRNA expression. The analysis performed also identified the marked effect that post‐exercise recovery strategies exert on the mechanisms of ribosome biogenesis. The majority of the measured signaling and transcriptional responses required for ribosome biogenesis were markedly suppressed in response to CWI immediately after the exercise bout. Thus, it is tempting to speculate that if post‐exercise ribosome biogenesis is regularly suppressed in response to CWI, then this could partly explain the smaller gains in both muscle mass and strength that accompany long‐term use of CWI during resistance training (Roberts et al. 2015).

Ribosome biogenesis is subject to complex regulation through multiple pathways (Kusnadi et al. 2015). Of the cellular signals for rRNA transcription, UBF is a central point of convergence. UBF possesses two splice variants, UBF1 and UBF2 (O'Mahony and Rothblum 1991). UBF2 is more abundant in quiescent cells. Although both UBF1 and UBF2 are responsive to a growth stimulus, UBF1 expression increases to a greater extent than UBF2 (Hisatake et al. 1991; Kuhn et al. 1994). Functionally, UBF1 is the de facto rDNA promoter transcription factor, whereas UBF2 has a lower stimulatory effect on rDNA transcription (Kuhn et al. 1994). Consistent with these functions, this study demonstrated that UBF1 protein expression increased during exercise recovery, with no change observed for UBF2.

The activation of UBF is regulated by the cell cycle regulator cyclin D1, which in turn is translationally regulated by MNK1‐eIF4E signaling (Mamane et al. 2004; Topisirovic et al. 2004). In the current study, RE strongly activated the MNK1‐eIF4E‐cyclin D1 axis. Phosphorylation of MNK1 Thr197 and its target eIF4E Ser209 were increased early after exercise (2 h), returning to basal levels of phosphorylation by 24 h post‐exercise. Consistent with this, p38, a major MNK1 kinase (Landon et al. 2014), was strongly phosphorylated 2 h after RE. This response was accompanied by increased expression of cyclin D1 protein (but not its mRNA) at the same time point, with the increased protein levels of cyclin D1 persisting for the duration of the post‐exercise recovery period (48 h). Because cyclin D1 has a short half‐life (Alao 2007), the sustained protein abundance cannot be solely due to eIF4E phosphorylation. The results of this study show that total eIF4E protein was also increased at later time points (24 and 48 h) of recovery. Previously, overexpression of eIF4E protein has been shown to increase cyclin D1 protein expression (Rosenwald et al. 1993). Thus, we speculate that the higher levels of cyclin D1 measured after 24 h and 48 h may be explained by the increased eIF4E protein abundance.

Cyclin D1 forms a dimeric active complex with CDK4, a kinase that phosphorylates and activates UBF (Voit et al. 1999). Phosphorylation of UBF is critical for rDNA transcription (O'Mahony et al. 1992; Voit et al. 1995), specifically at Ser388 and Ser484 (Voit et al. 1999; Ayrault et al. 2006). These UBF residues were highly phosphorylated at later time points of the recovery period following RE. UBF and TIF‐IA, along with a few other transcriptional factors of the preinitiation complex, recruit Pol I and promote rRNA synthesis (Russell and Zomerdijk 2005). TIF‐IA serves as a bridge between the Pol I and the preinitiation complex formed by UBF and SL1 (Russell and Zomerdijk 2005; White 2005). In the present study, TIF‐IA phosphorylation followed a pattern opposite to that of UBF, as has previously been described (Figueiredo et al. 2015). Following RE, there is a rapid phosphorylation of TIF‐IA at Ser649, as a result of the activation of the ERK pathway, which is the upstream kinase of TIF‐1A (Zhao et al. 2003; Figueiredo et al. 2015).

Some evidence suggests that Akt is also an important regulator of ribosome biogenesis (Chan et al. 2011; Nguyen le and Mitchell 2013). This led us to measure Akt phosphorylation at the phosphosite most related to its kinase activity (Thr308) (Moore et al. 2011; Vincent et al. 2011) and phosphorylation of its downstream target, PRAS40 at Thr246 (Vander Haar et al. 2007). Akt phosphorylation was unchanged up to 24 h after exercise. PRAS40 phosphorylation was lower than pre‐exercise 2 h after exercise, returning to pre‐exercise levels by 24 h. These results suggest that ribosome biogenesis may occur independently of Akt activity following RE – at least up to 24 h after exercise.

The greater expression of UBF, c‐Myc, and cyclin D1, in addition to UBF phosphorylation status, was matched by increased levels of rDNA transcription. All primer sequences used to perform real‐time PCR for pre‐rRNA (which span different portions of the 45S rRNA, namely 45S, ITS‐5.8S, ITS‐28S, and ETS‐18S) yielded similar results: 45S pre‐rRNA was markedly increased 24 h after exercise and remained elevated 48 h after exercise. These data are similar to those observed previously (Nader et al. 2014; Stec et al. 2015). Additionally, mRNA expression of Pol I factors (UBF, TIF‐IA and PolR1B) and the rRNA processing factor NIP7 were increased following RE. 45S rRNA undergoes cleavage and processing to form the mature ribosomal rRNA transcripts (5.8S, 18S, and 28S). However, there were no changes in the abundance of the mature rRNA transcript; thus, a repeated exercise stimulus may be necessary to enable increased mature cellular rRNA (Figueiredo et al. 2015). Collectively, these data suggest that RE promotes rDNA transcription, the expression of the Pol I regulon factors, and factors associated with rRNA processing.

**Figure 6:**
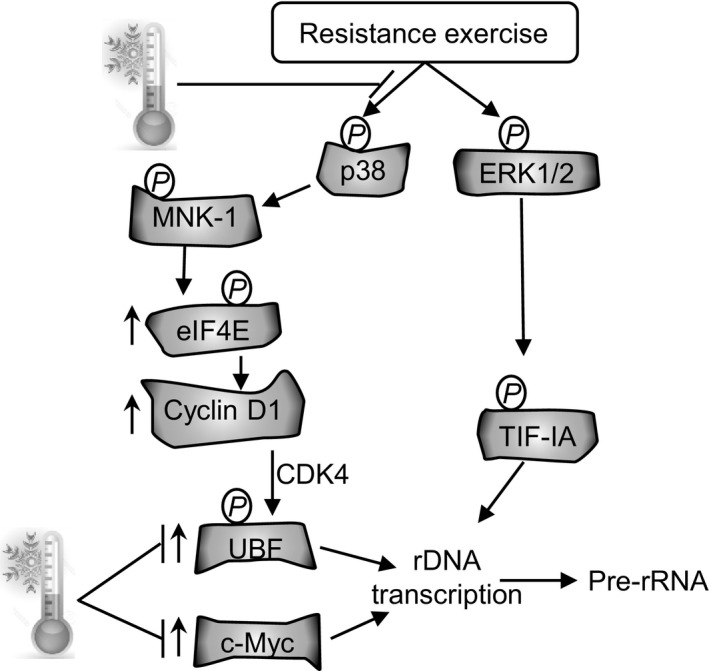
Schematic representation of the signaling pathway affecting rRNA synthesis following resistance exercise and where CWI might interfere with this pathway.

Repeated CWI following RE attenuates muscle hypertrophy and strength gains to chronic resistance training (Roberts et al. 2015). RE may promote gains in muscle mass and strength by activating stress signaling pathways. This effect is evidenced by the robust increase in the classical stress‐activated kinase, p38 MAPK (Obata et al. 2000; Coulthard et al. 2009), in the present study. CWI appears to attenuate this acute stress response to RE, which has downstream effects on many factors involved in ribosome biogenesis, including MNK1, eIF4E, UBF, cyclin D1, and c‐Myc protein (shown diagrammatically in Fig. 6). Previously, we proposed that the smaller gains in muscle mass and strength that we observed after 12 weeks of strength training combined with CWI could be due to acute attenuation of activation of satellite cells and kinases in the mTOR pathway (Roberts et al. 2015). The present data suggest that CWI may also attenuate adaptations to strength training by reducing acute activation of ribosome biogenesis after RE. The physiological/biochemical mechanisms by which CWI reduces activation of p38 MAPK and downstream factors involved in ribosome biogenesis are not known, but they may include changes in muscle temperature, blood flow, oxidative stress, and/or osmotic stress.

Our data demonstrate that resistance exercise leads to sustained activity of signaling pathways involved in ribosome biogenesis that persists for the 2 days of analysis. Importantly, we provide new insights into how these specific signaling pathways may contribute to mRNA translation of specific targets in skeletal muscle after exercise. These findings have important implications for future studies and for understanding the mechanisms underlying muscle hypertrophy. Resistance exercise appears to modulate these signaling pathways in a bimodal fashion. Initially, shortly after RE, there is robust activation of proteins (kinases and transcriptional and translational factors) that induce gene expression and mRNA translation of specific proteins upstream of the ribosome biogenesis machinery. During the later stages of postexercise recovery, ribosome biogenesis is initiated and the muscle protein synthesis capacity increases, supporting muscle growth. Another novel finding from this study is that CWI interferes with p38‐MNK1‐eIF4E axis and UBF and c‐Myc activation responses, which resulted in a blunted increase in pre‐rRNA synthesis promoted by RE. In the long term, this interference may contribute to smaller cumulative gains in muscle mass and strength in response to chronic resistance training.

## Conflict of Interest

None declared.
